# Usage Trends of Open Access and Local Journals: A Korean Case Study

**DOI:** 10.1371/journal.pone.0155843

**Published:** 2016-05-19

**Authors:** Jeong-Wook Seo, Hosik Chung, Jungmin Yun, Jin Young Park, Eunsun Park, Yuri Ahn

**Affiliations:** 1 Department of Pathology, Seoul National University College of Medicine, Seoul, South Korea; 2 Contents Platform Lab, Naver Corporation, Seongnam-si, Kyunggi-Do, South Korea; 3 Korea National Industrial Convergence Center, Korea Institute of Industrial Technology, Ansan-si, Kyunggi-do, South Korea; 4 Seoul National University Medical Library, Seoul, South Korea; Universidad de Las Palmas de Gran Canaria, SPAIN

## Abstract

Articles from open access and local journals are important resources for research in Korea and the usage trends of these articles are important indicators for the assessment of the current research practice. We analyzed an institutional collection of published papers from 1998 to 2014 authored by researchers from Seoul National University, and their references from papers published between 1998 and 2011. The published papers were collected from Web of Science or Scopus and were analyzed according to the proportion of articles from open access journals. Their cited references from published papers in Web of Science were analyzed according to the proportion of local (South Korean) or open access journals. The proportion of open access papers was relatively stable until 2006 (2.5 ~ 5.2% in Web of Science and 2.7 ~ 4.2% in Scopus), but then increased to 15.9% (Web of Science) or 18.5% (Scopus) in 2014. We analyzed 2,750,485 cited references from 52,295 published papers. We found that the overall proportion of cited articles from local journals was 1.8% and that for open access journals was 3.0%. Citations of open access articles have increased since 2006 to 4.1% in 2011, although the increase in open access article citations was less than for open access publications. The proportion of citations from local journals was even lower. We think that the publishing / citing mismatch is a term to describe this difference, which is an issue at Seoul National University, where the number of published papers at open access or local journals is increasing but the number of citations is not. The cause of this discrepancy is multi-factorial but the governmental / institutional policies, social / cultural issues and authors' citing behaviors will explain the mismatch. However, additional measures are also necessary, such as the development of an institutional citation database and improved search capabilities with respect to local and open access documents.

## Introduction

The increasing popularity of the internet has resulted in several changes to the use of the scholarly information. Journals now have easier publishing processes than in previous years, which has led to an increase in the number of international and local journal articles being published. These changes are beneficial for authors. However, it is now harder for the editors and publishers of local journals to collect good manuscripts from local research communities, particularly in less developed countries [1–3]. The second major change is that database technologies have been extensively applied to search engines and to link articles [4]. Scholarly databases such as Web of Science (WoS)[5] or Scopus[6] highlight premium research articles, and locally produced research articles from less developed countries receive fewer citations [2, 7, 8]. Additionally, open access (OA) articles have been integrated into digital publishing models.

Seoul National University (SNU) has a two-directional strategy regarding research papers. The university encourages publishing in international journals with higher impact factors, but also encourages publishing local research in local journals. South Korean journals are integrated into the local research environment and are valuable resources to junior or undergraduate students for language and educational purposes.

The WoS and Scopus citation databases are the most important resources for assessing researches [9–11]. Currently, citation databases are used mostly to determine the quality of journals (the impact factor) or articles that are most frequently cited. Database coverage is critical if we extend the analysis to different aspects of research (e.g., an author’s citing behavior or an assessment of the cited journals or papers). However, popular citation databases such as WoS and Scopus have limited coverage of local journals, non-English literature, and some subject areas (e.g., social sciences and humanities) [12–14]. This lack of coverage has been compensated for by using national citation databases in countries including China [15] and South Korea[7, 16, 17].

Open access is an initiative that intends to increase the access and application of scholarly information without barriers [18]. This initiative received global consensus at the Global Research Council in 2013 [19] and 2014 [20], and the OA initiative of Berlin [21]. However, controversy arises if OA articles are cited as often as subscription articles. A review of the citation advantage of OA articles from 31 previous studies found that 27 studies concluded that there are advantages and 4 found negative results [22]. Overall, the average citation rates for subscription journals were approximately 30% higher than non-subscription journals. However, the difference mostly disappeared when we controlled for the age of the journal and the location of the publisher, particularly for medicine and health journals [22, 23]. Papers from OA mandated institutions [8] and developing countries appeared to receive more citations in more recent years [24, 25], but an increase in citations is very difficult to measure. One of the studies with negative results found that there was a positive effect on the number of downloads, but no significant effect on the number of citations [26].

We generally understand that the usage trends of OA and local journals by SNU researchers reflect their current understanding of the value of the articles. However, it is possible that citation behavior can be biased towards articles from local or OA journals. We do not believe this bias is intentional, but if it exists, we should correct for it. Our objective is to answer the following questions.

- What is the proportion of OA articles in major scholarly databases? That is, we wish to determine the proportion of articles from OA journals among the total number of articles in these databases.
- What is the proportion of OA papers to all published papers that were authored by SNU researchers and indexed in the WoS database?
- What proportion of referenced articles from documents published by South Korean authors are from local or OA journals? Is the proportion of OA articles in the referenced articles similar to the proportion of OA articles in the database?

## Materials and Methods

### Journal databases and defining OA journals

We developed our journal lists from different online sources, that is, WoS [27], Scopus [28], Ulrich’s Global Serials Directory [29], the directory of OA journals (DOAJ) [30], PubMed database [31], BioMed Central (BMC) [32], and the Korean Citation Index (KCI) [33].

Open access manuscripts are labeled in DOAJ, PMC, WoS, Scopus, KCI, and BioMed Central (BMC) but not in Ulrich’s Global Serials Directory or PubMed. Some journals are defined differently by different sources, and some OA journals were previously subscription journals. We can use two strategies to determine the number of articles in OA journals using WoS or Scopus. One is to filter OA articles based on the OA journal label supplied by WoS. The other is to count articles based on our own definition of OA journals. This alternative approach is useful when journals are not listed in these databases. We define an OA journal as one that is defined as such in at least one of our sources (a list of OA journals in the WoS database is provided in S1–S10 Tables). Individual documents available as OA from hybrid journals, institutional repositories, or aggregators are not considered OA. Subscription journals are defined as not an OA journal.

We considered the journals listed in WoS in 2014. Published papers are defined as those in the WoS database. WoS cited papers are papers from a WoS journal listed in the 2014 database, regardless of when the cited papers were published.

We collected 256,054 journal titles with unique ISSN from different resources; 12,422 (S1 and S2 Tables) from WoS [27], 33,052 (S3 Table) from Scopus [28], 240,917(S4 Table) from Ulrich’s Global Serials Directory [29], 9,709 (S5 Table) from DOAJ [30], 25,269 (S6 and S7 Tables) from PubMed [31], 460 (S8 Table) from BMC [32], and 2,084 titles (S9 and S10 Tables) from KCI [33].

We defined a journal as local if the editing organization or institution was in South Korea. Some of these journals were published by international publishers with a foreign address, but they were still considered local. We calculated the proportion of cited papers from local journals for each year.

### Collecting documents from WoS or Scopus that were authored by SNU researchers

We found SNU documents in WoS database by filtering for addresses containing “Seoul Natl Univ” and for the year published in 1998–2014. This identified articles, domestic or foreign, by more than one author from SNU. We collected 88,485 articles from WoS (accessed March 2016). This reduced to 76,583 when we limited the document types to article, proceedings paper, review, and letter. Meeting abstract (10,462) was the most common type of excluded document. In this study, we used 76,569 articles for the proportion of OA articles in WoS.

We found SNU documents in Scopus database by filtering for affiliations of “Seoul National University, Seoul National University College of Medicine, Seoul National University Hospital or Seoul National University Bundang Hospital” and for the year published in 1998–2014. We collected 92,750 articles from Scopus (accessed March 2016). This reduced to 90,474 when we limited the document types to article, conference paper, review, and letter. In this study, we used 90,474 articles for the proportion of OA articles in Scopus.

### Analysis of journal names from the cited references

The ISSNs of the journal titles of cited references were identified by cross-referencing the name of the journal with our journal name database (journal names and their abbreviations are provided in the S1–S10 Tables). Because some journals have different abbreviations and WoS may contain insufficient or erroneous information, we could not identify all of the ISSNs (S11 Table).

In total, 2,750,485 cited papers were collected from 52,295 published papers authored by researchers from “Seoul Natl Univ” between 1998 and 2011. The proportion of articles for which we could determine the journal ISSNs was 80.8% of matched articles in 1998, 81.6% in 1999, 88.2% in 2010, and 88.6% in 2011 (Table 1, S12 Table). We matched the ISSNs of 3,029 journals from 55,143 papers cited in 1,665 papers that were published in 1998. This increased to 9,832 journals from 336,014 papers cited in 6,100 papers that were published in 2011.

**Table 1:** Relative proportions of different publishing statuses of papers in WoS cited between 1998 and 2011 by authors from Seoul National University.

Year	1998	1999	2000	2001	2002	2003	2004	2005	2006	2007	2008	2009	2010	2011
**Total No. published papers**	1,655	1,949	2,214	2,613	2,879	3,189	3,476	3,743	4,030	4,503	4,913	5,187	5,844	6,100
**Total No. cited papers**	68,246	81,768	97,816	117,203	133,651	153,111	169,141	189,108	207,098	233,017	277,394	296,526	347,158	379,248
**Average No of cited papers**	41	42	44	45	46	48	49	51	51	52	56	57	59	62
**% of matched articles**	80.8	81.6	82.4	82.3	83.7	84.3	85.0	86.2	86.3	86.9	88.0	87.5	88.2	88.6
**% Global Subscription SCI**	68.4	70.1	70.5	71.4	72.8	74.2	74.9	76.1	76.7	77.1	78.4	78.2	79.1	79.3
**% Global Subscription Non-SCI**	9.7	8.8	8.9	8.2	8.1	7.1	6.9	6.8	6.3	6.1	5.5	5.1	4.9	4.6
**% Global OA SCI**	1.4	1.5	1.5	1.5	1.5	1.5	1.8	1.7	1.8	2.1	2.4	2.5	2.7	3.1
**% Local Subscription SCI**	0.9	0.8	1.0	0.8	0.8	0.9	1.0	1.1	1.0	1.2	1.2	1.1	0.9	1.0
**% Local OA SCI**	0.3	0.3	0.3	0.2	0.3	0.3	0.3	0.3	0.3	0.3	0.3	0.4	0.4	0.5
**% Local Subscription Non-SCI**	0.2	0.2	0.2	0.2	0.1	0.2	0.2	0.2	0.1	0.1	0.2	0.1	0.2	0.1
**% Global OA Non-SCI**	0.0	0.0	0.0	0.0	0.0	0.0	0.0	0.0	0.0	0.0	0.0	0.0	0.0	0.0
**% Local OA Non-SCI**	0.0	0.0	0.0	0.0	0.0	0.0	0.0	0.0	0.0	0.0	0.0	0.0	0.0	0.0
**% Other**	19.2	18.4	17.6	17.7	16.3	15.7	15.0	13.8	13.7	13.1	12.0	12.5	11.8	11.4

Local, journals with a South Korean editing organization or institution; Global, journals other than local; OA, journals defined as open access in at least one of directory of open access journals (DOAJ), PMC, Web of Science (WoS), Scopus, BioMed Central (BMC), or Korean Citation Index (KCI); Subscription, journals other than OA; SCI, journals listed in Science Citation Index of WoS 2014; Non-SCI, journals other than SCI; Other, non-journal references or journal name could not be found in our database.

### Analyzing the usage trends of OA and local journal articles

We developed a database containing journal titles, published papers authored by SNU researchers, and references cited in these published papers. Using this database, we designed strategic plans for future research on OA and local journal issues. There were two steps in our analysis. The first step was to understand the available resources. The second was to determine the nature of this database and use this knowledge to design customized secondary resources.

In this paper, we use the term “published papers” for articles, proceedings papers, letters, and reviews by SNU authors and the term “cited papers” for papers cited by these published papers. This is to minimize the confusion between citing papers (“published papers”) and cited papers. Otherwise, we use terminology based on the citation typing ontology that a cited paper is “a reference within a particular citing work to another publication (e.g., a journal article, a book chapter, or a web page).” [34]

There are several intrinsic limitations to this research. The currently available resource is the biggest limitation. The heterogeneities of authors at institutional and journal levels represent an inevitable handicap, although the South Korean community is largely homogeneous. The intrinsic diversity of classifications of subject areas by different resources and a lack of a standard analysis protocol must also to be considered.

## Results

### Proportion of OA articles by SNU authors in WoS and Scopus

**Fig 1:**
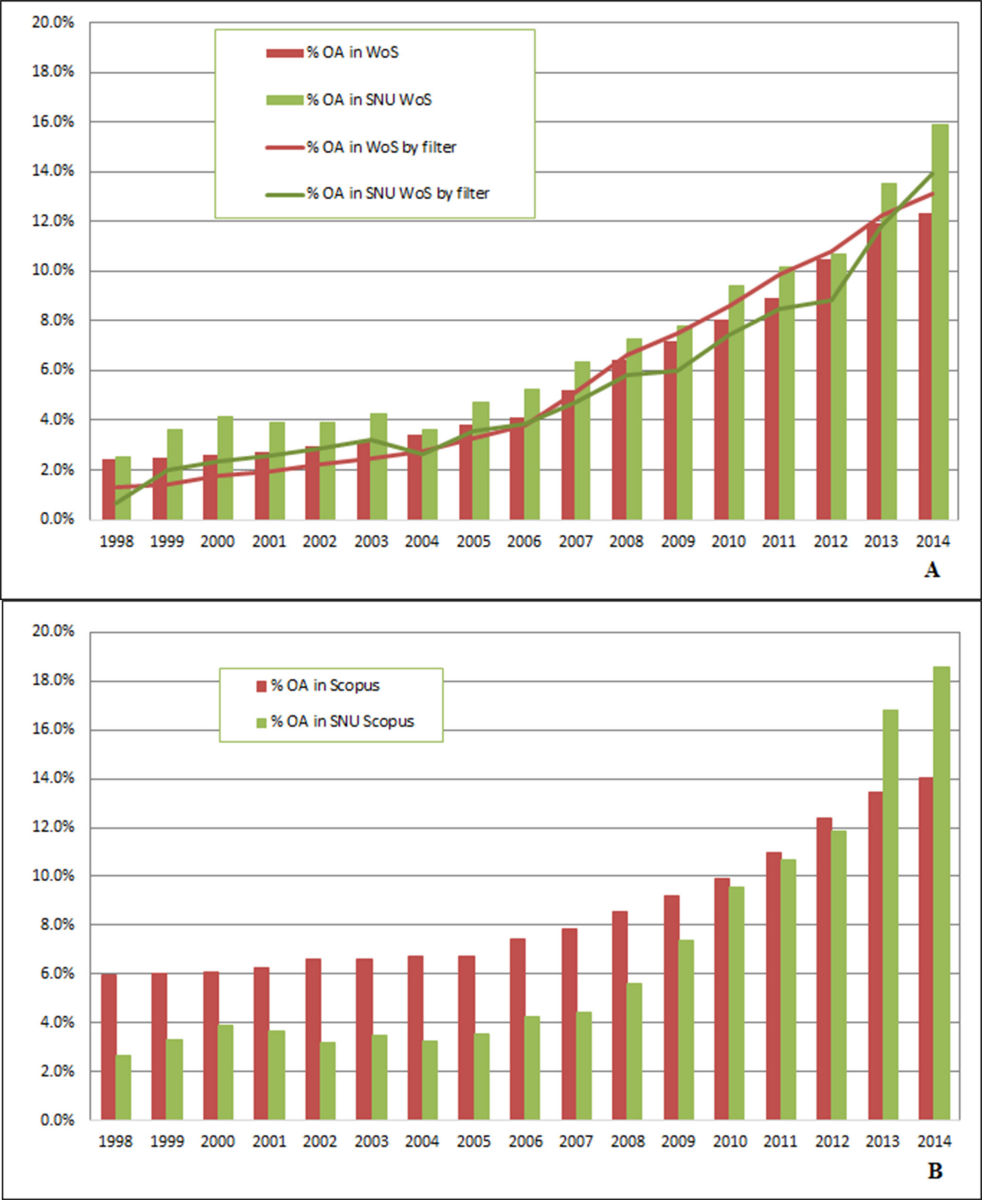
(A) Proportion of papers published in OA journals compared with all papers in WoS (red) from 1998 to 2014, and for papers from SNU (green). The bar chart is based on a fixed list of journals defined as OA in 2014, and the line graph is based on the OA filter supplied by WoS. (B) Proportion of papers published in OA journals compared with all the papers in Scopus (red) from 1998 to 2014, and for papers from SNU (green). The proportion is based on a fixed list of journals defined as OA in 2014.

Among 12,422 journal titles in the WoS databases accessed in June 2014, 1,216 titles (9.8%, S2 Table) were OA. The proportion of OA articles from OA titles in WoS in 2014 was 12.4% (191,875/1,552,450 articles). The proportion of OA articles in WoS for each year between 1998 and 2014 is shown in Fig 1 (S13 and S14 Tables). Fig 1A shows the proportions calculated using two methods: using our definition of OA journals defined in 2014 (bar chart), and using the WoS definition (line chart). The science citation database (extended) had a higher rate of OA (SCI(E); 982/8,040 or 12.2%) than the social science database (SSCI; 165/2,986 or 11.1%) or the arts and humanities database (A&HCI; 69/1,396 or 4.9%) (S14 Table).

The Scopus database (accessed June 2014) contained 2,583 (12.4%) OA journal titles among a total of 20,874. We calculated the proportion of OA articles in Scopus based on our fixed definition (Fig 1B) but Scopus does not have OA filter. The OA proportion in Scopus was 17.7% (758/4,290) for life sciences journals, 15.9% (1,056/6,656) for health sciences, 11.4% (762/6,683) for physical sciences, 7.5% (554/7,394) for social sciences, and 5.8% (143/2,445) for arts and humanities (S14 Table).

Our analysis of 76,569 published papers authored by SNU researchers in WoS during 1998–2014 revealed that 6,381 (8.3%) papers were published in OA journals, when using our definition of OA journals defined in 2014 (bar chart in Fig 1A). We also used the OA filter supplied by WoS (line chart in Fig 1A). The proportion of published papers from OA journals by SNU authors using these two methods was stationary until 2006 (5.2%), but increased to 15.9% in 2014 (Fig 1A).

A same calculation for the proportion of OA papers in Scopus based on 90,474 published papers authored by SNU researchers in Scopus during 1998–2014 revealed that 7,907 (8.7%) papers were published in OA journals (bar chart in Fig 1B). The proportion of published papers from OA journals by SNU authors in Scopus was stationary until 2007 (4.5%), but increased to 18.5% in 2014 (Fig 1B).

### Analysis of references in papers published by SNU researchers in WoS

There were 68,246 cited papers in 1998, and 80.8% could be matched with a journal ISSN (Table 1). There were 46,660 (84.6% of matched references) from global subscription SCI journals. There were 379,248 cited papers in 2011, and 88.6% could be matched with a journal ISSN. There were 300,729 (89.5% of matched references) from global subscription SCI journals (Table 1). The average number of cited papers for each paper in 1998 was 41 but the number increased to 62 in 2011.

The proportion of cited papers from local journals was 1.7% in 1998 and 1.5% in 1999, and it remained at this approximate level until 2011 (1.7% in 2010 and 1.8% in 2011) (Table 2). However, the proportion of papers from OA journals increased from 2.1% in 1998 and 2.2% in 1999 to 3.5% in 2010 and 4.1% in 2011 (Table 2). The combined proportion of OA and local journals was 3.4% in 1998, 2.4% in 1999, 4.8% in 2010, and 5.3% in 2011 (Table 2).

**Table 2:** Trends in the proportion of cited papers from subscription SCI journals (local or global), OA journals (local or global), local or OA journals, and local journals (OA or subscription) among all referenced articles with matched ISSNs.

Year	1998	1999	2000	2001	2002	2003	2004	2005	2006	2007	2008	2009	2010	2011	Total
**% Subscription SCI**	85.7	86.8	86.7	87.7	87.9	89.2	89.3	89.5	90.1	90.1	90.4	90.6	90.8	90.6	89.7
**% OA + Local**	3.4	3.4	3.6	3.3	3.4	3.5	3.8	3.8	3.8	4.3	4.7	4.8	4.8	5.3	4.3
**% OA**	2.1	2.2	2.2	2.1	2.2	2.1	2.4	2.3	2.5	2.7	3.2	3.4	3.5	4.1	3.0
**% Local**	1.7	1.5	1.8	1.5	1.5	1.7	1.7	1.8	1.7	1.9	1.9	1.9	1.7	1.8	1.8

Subscription SCI, combined data from local or global SCI journal articles; OA + Local, combined data for OA local/global and any types of local journals (OA/subscription, SCI/non-SCI); OA, combined data for any type of OA (local/global, SCI/non-SCI); Local, combined data for any type of local journal (OA/subscription, SCI/non-SCI).

The proportion of articles from subscription journals was 98.0% in 1998, 97.8% in 1999, 96.5% in 2010, and 95.9% in 2011. However, the proportion of subscription SCI journals increased from 85.7% in 1998 to 86.8% in 1999 and then plateaued, with 90.8% in 2010 and 90.6% in 2011 (Table 2).

**Fig 2:**
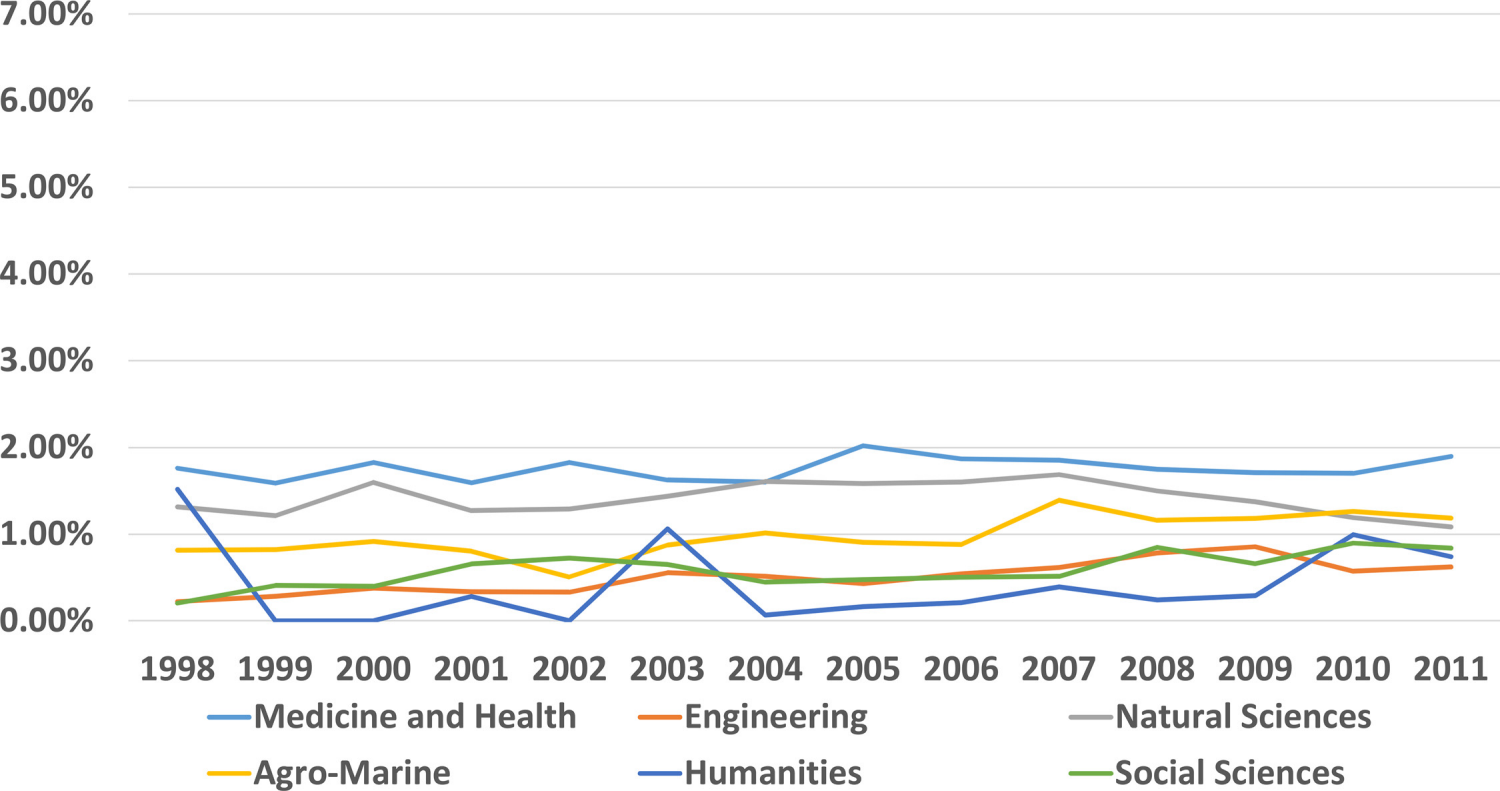
Proportions of cited papers from local journals for each year and subject area.

**Fig 3:**
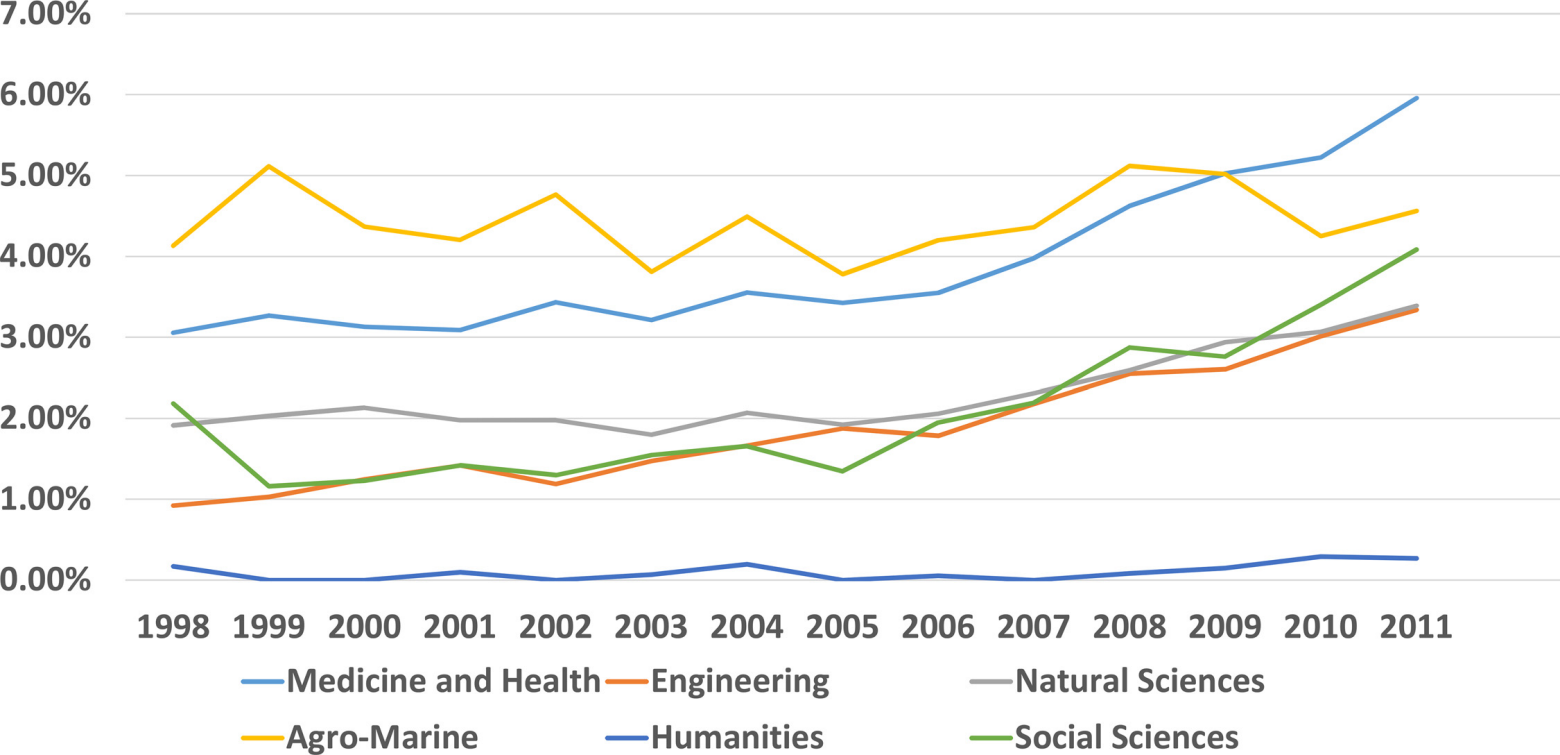
Proportion of cited papers from OA journals for each year and subject area.

We analyzed the proportion of cited papers from local and OA journals with respect to their subject areas (Figs 2 and 3, S15 Table). The proportion of papers from local journals was low and relatively constant. There were more locally produced papers on medicine and health, and natural sciences than the other subject areas (S1 Fig). There was a large proportion of papers from OA journals among the cited papers from agriculture and marine sciences (4.1% in 1998 and 4.6% in 2011). The proportion for papers from medicine and health areas was also high (3.1% in 1998 and 6.0% in 2011). There were increasing trends in the use of papers from OA journals in social sciences, natural sciences, and engineering (Fig 3).

### Citation trends for OA and local journal articles

Our assessment of the available resources (WoS and Scopus) for our analysis of citation trends was partly positive. They had stable selection processes of covering journal titles. Search functions are very well designed and systematic. We could analyze research papers using WoS or Scopus by accessing the relevant articles and groups of papers. It was not possible, however, to compare data between these two databases. They adopt different classification and definitions of the metadata system which are quite reasonable on their own individuals. So we found it better to choose one of the two rather than to use both of them.

There were other limitations for our use of these resources. The biggest challenge was to modify the database to meet our researchers’ demands. Our hope was to change the coverage of the database so that we could analyze the citation trends among articles published in local journals or among articles published by local authors. We therefore thought necessary to develop a secondary citation database from the existing databases, constructing a customized secondary resource on papers authored by researchers in our institution.

**Fig 4:**
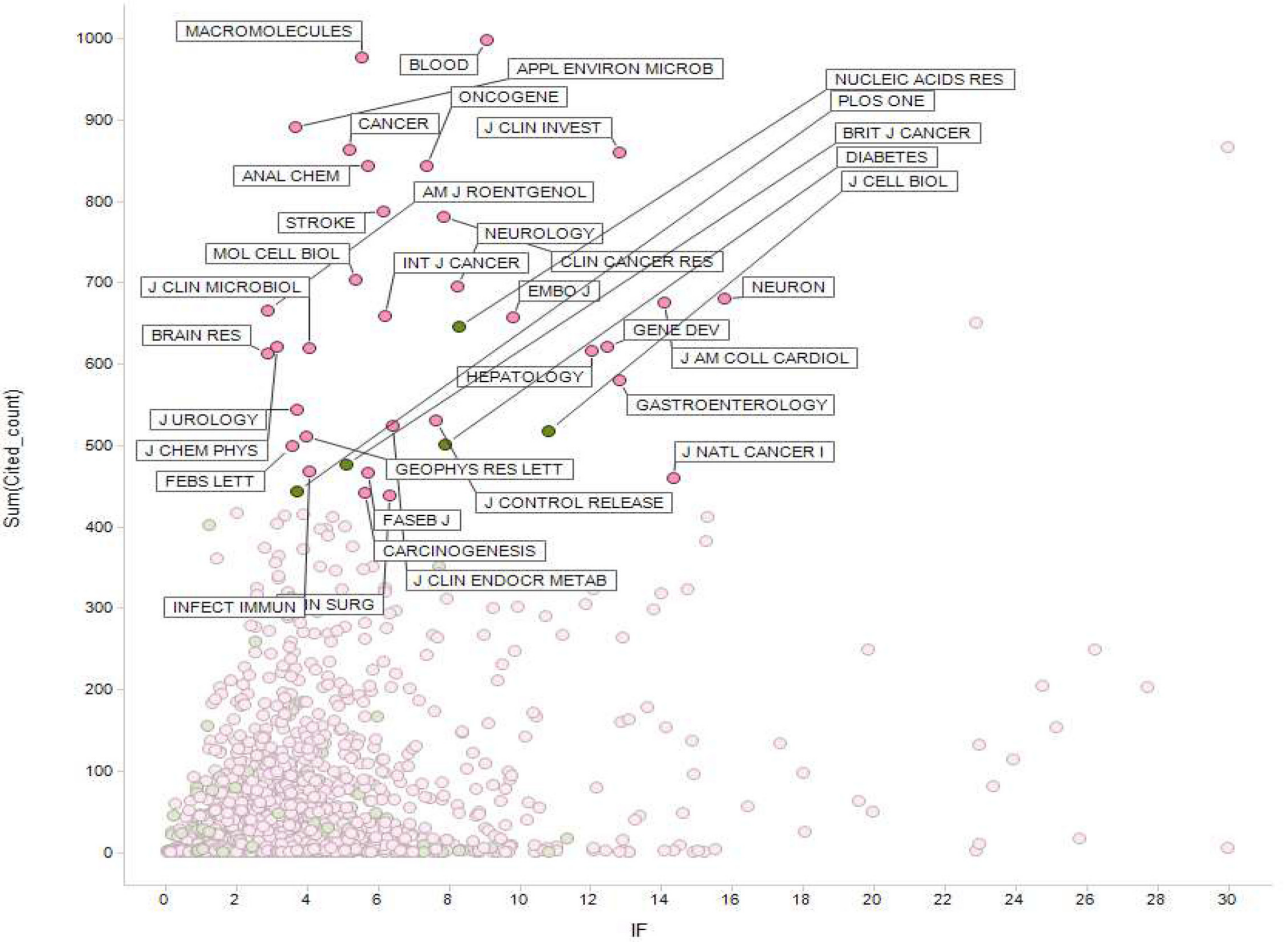
Citation frequencies from our database (y-axis) compared with the impact factor based on the WoS database (x-axis), for medicine and health journals (2010–2011). Frequently cited journals are marked with their names. Pink dots represent subscription-based journals and green dots are OA journals.

Nonetheless, we could make a few interesting output of the current database. One was to make relevant lists of highly cited journal titles, by subject area and year, and then correlate the citation frequency with journal impact factors in the WoS database so that we could determine journals that were frequently cited by South Korean researchers (Fig 4).

## Discussion

We believe that issues related to local and OA journals are important to the research community of South Korea. A small number of local articles in the international database has been considered an indicator of a low level of scientific activity in developing countries [35, 36] and non-English speaking countries [37]. There is another important group of local articles that are published in local journals which may not be included in the international database. These local articles are important for the assessment of the research and for developing their own research in certain countries. The main barriers to increasing the number of local articles are that local authors find it difficult to write papers and local journals are not always available in search databases. Based on economic criteria alone, South Korea is a developed nation, but is still a developing nation in terms of the publication of local scholarly articles [38]. In this paper, we studied the trends in the use of papers or numbers of cited papers from local journals.

Our overview of OA articles from a global perspective showed that, in 2014, OA journals accounted for 9.8% of journal titles in WoS and 12.4% in Scopus (S14 Table). In terms of the proportion of articles, there were 9.0% in WoS and 10.2% in Scopus, which is slightly lower than the proportion of journal titles. We have not assessed how many OA articles are used compared with the subscription-based articles.

Our comparison of the proportions of OA articles in WoS and Scopus suggests that there are some differences (Fig 1A and 1B). Scopus has higher proportion of OA articles than WoS. The two methods for determining OA articles did not produce significantly different results when applied to WoS data. Comparing the SNU proportions with the worldwide results, we found that the proportion of OA SNU papers was lower, but is now increasing. We also compared the proportion of SNU articles with those from Spain[39]. The proportions of OA papers in WoS from SNU, Spain, and the world were, respectively, 4.7%, 4%, and 2% in 2005, 7.3%, 8%, and 5% in 2008, 10.2%, 10%, and 7% in 2011, and 15.9%, 13%, and 10% in 2014[39]. Thus, research articles by South Korean authors were published by OA journals more often than articles by authors from other countries.

In contrast to the number of articles published in OA journals, the proportions of cited OA articles were significantly less. Most cited articles were published in global subscription journals. The contribution of OA or local journal articles was minimal. The number of cited OA articles increased from 2.1% in 1998 to 4.1% in 2011. Moreover, there has recently been a relatively constant increase in the use of OA articles (Fig 3). The proportion of cited articles from OA journals was more than 4% for agriculture and marine sciences, and medicine and health. Natural sciences, engineering, and social sciences had similar patterns and growth. The number of cited articles from local journals was disappointing. Less than 2% were cited in 1998 and the figure did not change until 2011. There is an increase in the number of manuscripts published by South Korean researchers, but they are not cited in other published documents. Medicine and health, natural sciences, and agricultural/marine sciences cite a somewhat higher proportion of local research. This probably reflects the influences of the local environment on this research, but the proportion is still very low (Fig 2). It is necessary to consider local and OA articles together because most of local SCI journals are open accessed (Table 2).

A previous study showed that the average citation rates were 30% higher for subscription journals than OA journals [23]. We can expect that 6.4% of cited references will be OA based on a very crude calculation with 30% lower citation rate for OA articles and 9% share ((9%×0.7) / (9%×0.7 + 91%) = 0.064). The proportion of OA articles (4.1% in Table 1) among references of papers by SNU authors excluding others (11.4% in Table 1) in 2011 is 4.6% (4.1% / (100% − 11,4%) = 0.046), which is still much lower than expected share. If we make a simple extrapolation of this data, we can expect that 11.7% of cited references will be OA based in 2014 ((15.9%×0.7) / (15.9%×0.7 + 84.1%) = 0.117).

The disciplines, age of the journals, and locations of the publishers have major influences of the different proportions [23]. The proportions for non-SCI subscription journals depend on the subject area. The proportion of non-SCI subscription journals in the social sciences is 28% with respect to journal titles, and 29% with respect to the number of cited articles. The proportion for medicine and health is similar to natural sciences; non-SCI subscription journals have proportions of 15–11% with respect to titles and 4–5% with respect to cited articles. Engineering, humanities, agriculture, and marine sciences have lower proportions.

There are certain caveats to this research. One major source of error in our calculations was the definitions of local, OA, and subscription journals. We tried to match the journal titles of cited articles with an ISSN so that we could determine if they were OA, SCI, or local journals. Our success rate using this method was 80.8% in 1998, and increased to 88.6% in 2011. Therefore, there is a 10–20% chance of error. This subset of unmatched, cited articles is generally considered to be non-serial articles such as monographs or gray literature. Journal titles that are not listed in SCI may contribute to these unmatched articles, but their contribution will be very small. We believe that the figures of SCI journals would not change if we increased these matching rates, but we expect increases for global non-SCI journals with OA or subscription-based access. The OA articles in this analysis do not include green road articles (institutional repositories).

There are several ways to speculate reasons why local or OA documents are not cited. One explanation is the “vicious cycle of academic research”: Researchers become discouraged if their work is not frequently cited, which may result in less enthusiasm to conduct future research, which reduces the number of published papers, which causes less of their work to be available in search databases, and thus further reduces their citations. This is a common issue in most countries with poor researches. The better explanation of this discrepancy is the “publishing/citing mismatch”. The increase of SNU’s research output is significant and the proportion of OA articles is also increasing, but articles published in local or OA journals are not used in cited references. The increase of published papers in subscription journals, OA journals or local journals is a good indicator of research development. If this increase in publication is not accompanied by a similar increase in cited papers from these publication groups, there is the publication/citation mismatch and we should find reasons and solutions of this mismatch.

Many countries invest research funds to support local research and apply policies on academic development. For example, a mandatory requirement to publish a set number of papers in journals listed in WoS. The outcome of this policy is often disappointing and produces several adverse effects. As previously mentioned, international journals publish more articles in each issue and new journals are founded to accept manuscripts that were previously rejected. Local journals receive fewer submissions and manuscript quality declines.

The gap between publishing and citing behaviors is significant regarding OA and local journals when we consider SNU researchers. Researchers may insist that they should cite only when they think it is valuable, and they should not be pushed to favor citing OA or local journals. Alternatively, this gap could be caused by South Korean researchers discriminating against OA and local journals, and instead preferring articles from prestigious journals that have high impact factors. This bias is an effort to show (to reviewers and readers) that their research is more relevant [10, 40].

We propose some solutions that may reconcile the publishing/citing mismatch, and increase citations from OA and local journals. The first action should be to find any issues of the policy on research promotion by the government or the institution. The second will be to find any social or cultural issues to discourage citations of articles published in local or OA journals. The third is to encourage researchers to increase their use and citations of articles from local and OA journals. However, we must also improve the situation by supplying better search engines and access to the relevant articles. That is, we need aggregated, searchable (abstract/citation) databases for articles from OA and local journals, which are similar to the database used in this research. Analysis of citation patterns based on a database with a customized document coverage is the key to our research. A citation analysis is always dependent on database coverage, and the objective of such an analysis depends on the particular group of documents we are interested in.

Editors of local journals strive to have their journals indexed in major databases but they are rarely successful [2]. Therefore, we think that it is important to develop regional databases covering local and regional information from local and regional journals [41–43]. Article metadata from local journals and citation databases are being aggregated in China [15] Japan [44] Latin America [45], Africa [46], Spain [47], India [48], Thailand [49], and South Korea [16, 17]. However, the quality and sustainability of these projects are unknown. KoreaMed is being developed for South Korean medical journals, and WPRIM (Western Pacific Region Index Medicus) is being developed for medical and health journals from the Western Pacific region of the World Health Organization. These could be models for abstract databases but the development of regional citation databases is still a challenging issue.

There is no doubt about the general benefits of internet, especially for researchers from less developed countries. The aggregated search processes of Google Scholar [50] and Naver Academic Services [51] have a tremendous role in improving access to research. These benefits, however, can increase the gap between developed and developing countries [3]. We believe that less developed and recently developed countries share similar concerns to South Korea, with respect to these issues.

## Supporting Information

S1 Fig Pie charts on contributions of different publishing types. (TIF)

S1 Table Journal title list SCI SSCI AHCI ISSN. (XLSX)

S2 Table OA journal title list in WoS. (XLSX)

S3 Table Journal title list Scopus. (XLSX)

S4 Table Journal Ulrichs Universe title list. (XLSX)

S5 Table Journal title list doaj. (XLSX)

S6 Table Journal title list Entrez. (XLSX)

S7 Table Journal title list PMC. (XLSX)

S8 Table Journal title list BioMed Central & SpringerOpen. (XLSX)

S9 Table Journal title list Korean Citation Index. (XLS)

S10 Table Journal title list Korean Citation Index Candidates. (XLS)

S11 Table Sample raw data for cited paper analysis. (XLSX)

S12 Table For Tables 1 and 2. (XLSX)

S13 Table For Fig 1A and 1B WoS, Scopus OA papers count. (XLSX)

S14 Table Journal analysis SCI SSCI AHCI Scopus OAJ. (XLSX)

S15 Table For Figs 2 and 3. (XLSX)
