# Supplementary figures and images for: Usage Trends of Open Access and Local Journals: A Korean Case Study
Source: PLoS One. 2016 May 19;11(5):e0155843. doi: 10.1371/journal.pone.0155843 (PMC4873189; doi:10.1371/journal.pone.0155843)

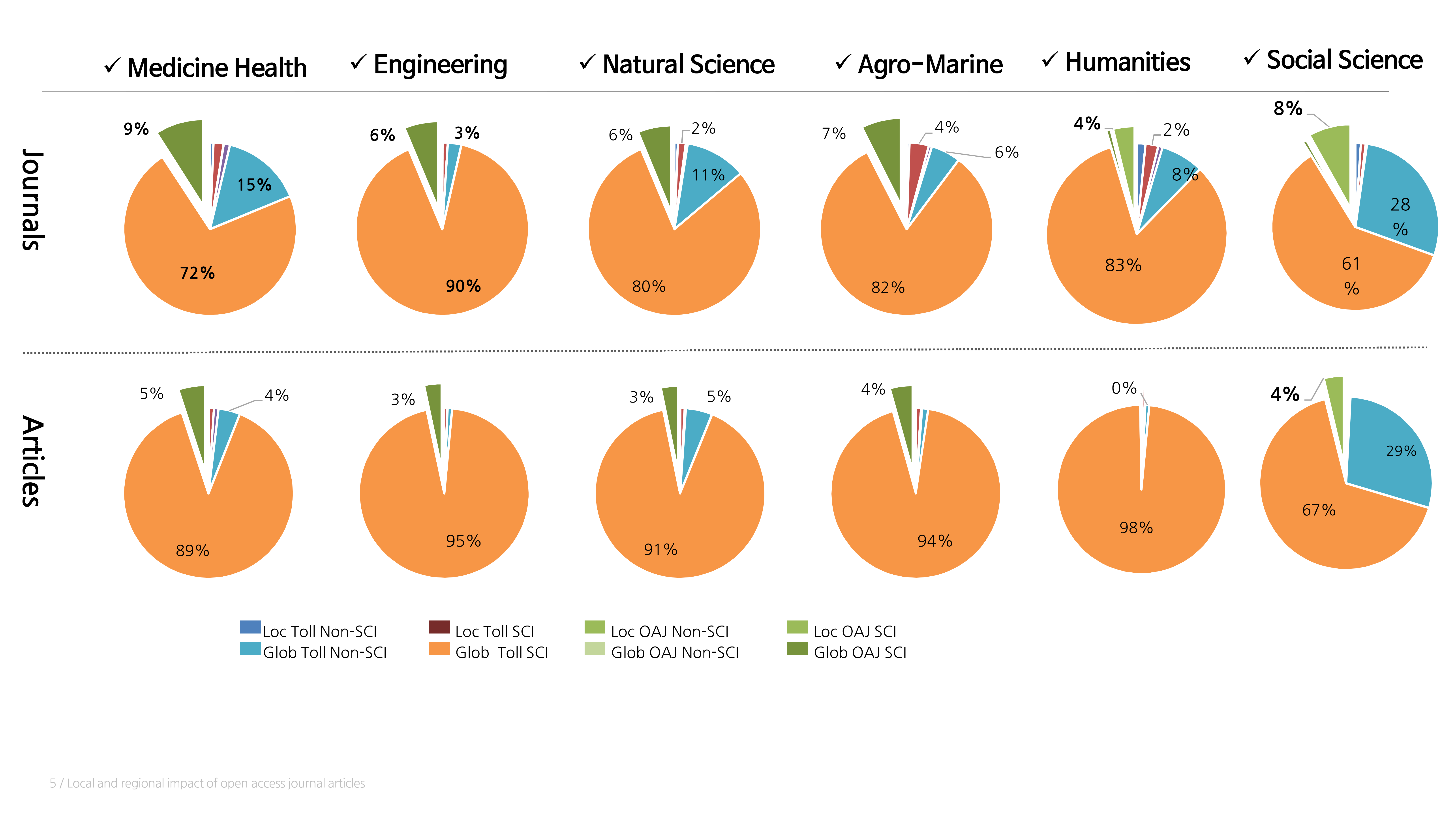

Supplement: S1 Fig — (TIF) [file pone.0155843.s001.tif]
